# Acetaminophen: A Liver Killer or Thriller

**DOI:** 10.7759/cureus.47071

**Published:** 2023-10-15

**Authors:** George S Zacharia, Anu Jacob

**Affiliations:** 1 Gastroenterology and Hepatology, Al Ahalia Hospital Mussafah, Abu Dhabi, ARE; 2 Anesthesiology and Perioperative Medicine, Al Ahalia Hospital Mussafah, Abu Dhabi, ARE

**Keywords:** antipyretics in liver disease, analgesic safety in liver disease, heptotoxicity, paracetamol, acetaminophen

## Abstract

Acetaminophen, or paracetamol, ranks among the most extensively utilized analgesic and antipyretic medications globally. The administration of acetaminophen to individuals with underlying liver disease has long sparked concerns regarding the potential risk of hepatotoxicity. However, the available literature and recommendations consider it a safe option in all forms of liver diseases and is deemed safe when used at recommended doses. This article aims to offer a concise review of the pharmacokinetics, toxicity profile, and the intricate considerations surrounding the safety of acetaminophen in patients with liver disease. By delving into the liver-acetaminophen interactions, we seek to provide a nuanced perspective on the use of acetaminophen in this critical patient population.

## Introduction and background

Acetaminophen, readily available over the counter, remains a popular choice for pain and fever management worldwide. The relationship between acetaminophen and the liver is intricate and multi-faceted. On one hand, acetaminophen stands as one of the leading causes of liver failure, while, on the other, it remains one of the preferred medications for alleviating fever and pain, even among individuals with liver conditions. However, using acetaminophen in the context of liver disease demands careful consideration. Given the liver's central role in metabolizing acetaminophen, it becomes particularly susceptible to toxicity when misused or administered to individuals with compromised liver function. This complex interplay underscores the need for cautious and informed approaches to acetaminophen usage in individuals with liver-related concerns.

## Review

Pharmacokinetics of acetaminophen and liver disease

Acetaminophen is primarily metabolized by hepatic enzymes to non-toxic metabolites. However, a small fraction undergoes cytochrome P450-mediated conversion to a highly reactive intermediate, N-acetyl-p-benzoquinone imine (NAPQI). Under normal circumstances, NAPQI is efficiently conjugated with glutathione and excreted harmlessly. In overdose or when the glutathione supply is depleted, NAPQI can overwhelm the detoxification pathways, leading to hepatocellular injury [1].

Alcohol exacerbates acetaminophen (paracetamol) hepatotoxicity by multiple mechanisms. Chronic alcohol consumption depletes hepatic glutathione, which helps neutralize NAPQI [2]. Chronic alcohol use can induce cytochrome P450 enzymes in the liver, specifically, CYP2E1, facilitating an increased conversion of acetaminophen to NAPQI [3]. Alcohol can contribute to a heightened inflammatory response in the liver. Acetaminophen-induced liver injury triggers an inflammatory cascade, and alcohol can exacerbate this process [4]. Chronic alcohol consumption impairs the regenerative capacity of the liver, leading to delayed recovery and potentially more severe liver injury [5].

Drugs that can induce hepatic cytochrome P450 enzymes potentially increase the production of toxic paracetamol metabolites and hepatoxicity. Concomitant use of rifampicin, isoniazid, phenobarbital, phenytoin, and St. John’s wort enhances the risk of acetaminophen hepatotoxicity [6-9]. Severe malnutrition or protracted fasting can deplete hepatic glutathione levels, making it more susceptible to paracetamol-induced hepatotoxicity [10]. Clinicians and patients should be cautious when using combination products containing acetaminophen, as it may lead to unintentional acetaminophen overdose if other medications are consumed concurrently.

Acetaminophen, “a killer of the liver”

Acetaminophen hepatotoxicity is dose-dependent, with risk increasing significantly when taken in doses exceeding 4,000mg daily in adults. Risk is heightened by malnutrition, prolonged fasting, alcohol consumption, use of enzyme-inducing medications, and underlying liver disease. Acute liver failure (ALF) develops only in 1%-2% of acetaminophen users; still, acetaminophen toxicity is the leading cause of ALF in developed countries [11]. Acetaminophen overdosages may be intentional, with suicidal ideation, and are more common in patients with underlying psychiatric diseases. Unintentional overdosage is equally shared and often results from using multiple acetaminophen-containing preparations, common in patients with polysubstance abuse. The use of acetaminophen, unaware of the underlying liver disease, also contributes to unintentional hepatotoxicity [12].

The severity of acetaminophen hepatotoxicity can vary widely, depending on the ingested dose of acetaminophen and the individual's susceptibility. In milder cases, individuals may remain asymptomatic. Symptoms usually manifest within 12 to 24 hours after ingestion, including nausea, vomiting, and right upper abdominal pain. Laboratory tests commonly reveal a substantial elevation in liver enzymes, such as alanine aminotransferase (ALT) and aspartate aminotransferase (AST), often reaching levels in the thousands and hyperbilirubinemia. However, in severe instances, patients may progress to fulminant liver failure, marked by hepatic encephalopathy, coagulopathy, and multiorgan dysfunction. In such dire circumstances, liver transplantation may become necessary to prevent the inevitable outcome of death [13].

Early recognition and treatment of acetaminophen are crucial and remain the critical determinant of outcome. Activated charcoal may be administered within the first hour of ingestion to help reduce absorption of the drug from the gastrointestinal tract. The efficacy of activated charcoal beyond two hours of ingestion is limited [14]. The Rumack-Matthew Nomogram, a plot of acetaminophen level to time since ingestion, determines the risk of developing hepatotoxicity and whether N-acetylcysteine (NAC) treatment is warranted. NAC is the cornerstone of acetaminophen overdose treatment. It replenishes hepatic glutathione stores, essential for detoxifying the toxic metabolite NAPQI.

An intravenous regimen is recommended for patients with severe overdose who cannot tolerate oral NAC, or when rapid treatment is required [15], with a loading dose of 150mg/kg over 15-60 minutes and a maintenance dose of 50mg/kg over four hours and 100mg/kg over 16 hours.

Oral therapy is effective when administered within eight hours of ingestion and is often used for patients at lower risk of severe hepatotoxicity [16], with a loading dose of 140mg/kg, given as a single dose, and a maintenance dose of 70mg/kg every four hours for 17 doses.

The King's College Criteria assesses the severity of acetaminophen overdose and predicts the risk of progression to acute liver failure. In cases of acute hepatic failure, liver transplantation may be considered.

Acetaminophen, “a thriller in liver disease”

Analgesic medications, including acetaminophen, non-steroidal anti-inflammatory drugs (NSAIDs), cyclooxygenase 2 inhibitors, opioids, anticonvulsants, and antidepressants, are primarily metabolized by the liver. Consequently, none of these medications can be considered entirely safe for individuals with liver disease. Also, no large-scale, well-designed trials have assessed analgesic safety in liver disease.

Despite its infamy as a potential hepatotoxin, acetaminophen assumes a lesser-known role as a potential ally in specific liver disease contexts. If dosed correctly, several short-term, low-volume trials suggest acetaminophen's safety in cirrhosis. Benson et al., 1983, evaluated acetaminophen at a dose of 4g/day for 14 days in patients with chronic stable liver disease, and it was well tolerated without adverse effects [17]. According to McGill et al., short-term, low-dose acetaminophen (650mg, twice daily, for less than one week) is safe in compensated cirrhosis [18]. A case-control study involving 170 patients determined that the use of acetaminophen at adjusted doses ranging from 2-3g per day was not associated with hepatic decompensation [19]. A randomized control trial by Dargère et al., with acetaminophen (1g thrice daily) in patients with hepatitis C virus infection, revealed no significant change in liver assays [20]. Dart et al. evaluated acetaminophen at a dose of 1g four times a day, in patients with chronic alcohol consumption with similar safe results [21]. Reviewing the published data, Lewis et al. [22] concluded that acetaminophen is non-toxic in cirrhotic patients at 2g/day dose. In a similar review published in the British Journal of Clinical Pharmacology, the authors concluded that acetaminophen is a safe and effective first-line agent in patients with liver diseases, irrespective of the etiology [23]. Another systematic review by Alavian et al. [24] concluded that acetaminophen is safe in most liver diseases at adjusted dosage. Acetaminophen is also deemed a safe analgesic in patients undergoing liver resection [25]. Unlike other analgesics, acetaminophen is not associated with sedation, nephrotoxicity, gastrointestinal bleeding, and platelet dysfunction. Considering the extensive available safety data, when used at appropriate dosage (up to 2-3g/day), acetaminophen should be the preferred first-line analgesic, antipyretic medication in patients with all forms of liver disease. However, research suggests limited awareness among clinicians and patients regarding acetaminophen's role in liver disease [26,27]. In a study encompassing over 2,000 healthcare providers, 40% of the participants were reluctant to endorse acetaminophen usage in patients with compensated cirrhosis. Equally intriguing, in the same study, physicians preferred recommending NSAIDs over acetaminophen in individuals with underlying liver disorders [27].

NSAIDs are particularly discouraged in patients with liver disease, especially in cases of cirrhosis, due to their heightened potential for causing kidney problems, gastrointestinal issues, and impairing platelet function [26-29]. NSAID-induced idiosyncratic hepatotoxicity has been extensively documented as well [27]. When it comes to cyclooxygenase 2 (COX-2) inhibitors, they may offer some protection against gastrointestinal bleeding compared to traditional NSAIDs. However, they carry an increased risk of cardiovascular side effects. Initial data suggests that patients with cirrhosis and ascites treated with celecoxib may experience decreased glomerular filtration rates. Therefore, the safety of COX-2 inhibitors in patients with cirrhosis necessitates further investigation [30]. Considering that opioids are primarily metabolized in the liver, it's important to note that patients with cirrhosis may experience reduced drug clearance and/or increased oral absorption, leading to the accumulation of opioids in the body. Opiates can induce sedation and potentially trigger hepatic encephalopathy, making them less desirable for patients with cirrhosis, particularly those with portal hypertension and encephalopathy. In cases where opioids are necessary for pain management, using lower doses and longer intervals between administrations is crucial to minimize associated risks. Hydromorphone, fentanyl, and tramadol may be preferable options in these scenarios [31-33]. Tricyclic antidepressants and anticonvulsants play a role in managing neuropathic pain. Tricyclic antidepressants undergo hepatic biotransformation with first-pass effects and are eliminated through the kidneys, necessitating dose adjustments in individuals with cirrhosis. Carbamazepine has been associated with hepatotoxicity, potentially leading to a swift deterioration in cirrhotic patients, and therefore, it should be avoided. Gabapentin and pregabalin, in contrast, do not rely on hepatic metabolism. Consequently, they emerge as attractive choices for managing neuropathic pain in patients with cirrhosis [34].

Acetaminophen dosing in liver disease

Alterations in drug metabolism, distribution, and elimination can significantly affect paracetamol pharmacokinetics, necessitating meticulous dosing adjustments to prevent hepatotoxicity. The National Institute for Health and Care Excellence (NICE) recommends a maximum daily dose of 2,000mg for cirrhosis. The American College of Gastroenterology (ACG) suggests 2,000mg daily, or even less, for severe liver disease [35]. The American Liver Foundation 2006 recommended daily acetaminophen not exceeding 3g for any prolonged period [36]. In 2009, the American Geriatric Society recommended no more than 2-3g of acetaminophen daily in older patients with hepatic insufficiency or a history of alcohol abuse [37]. Multiple studies also have concluded the safety of acetaminophen in liver disease at a dose of 2-3g daily [19,38,39]. The practice in National Health Services, Leeds Teaching Hospitals, is the reduction of the maximum dose of oral paracetamol to 3g in 24 hours in malnourished patients or Child-Pugh C cirrhosis patients [40]. In summary, acetaminophen may be used at 2-3g per day in patients with liver disease [41-43]. Frequent monitoring of liver function tests is advisable when acetaminophen is used in individuals with liver disease, to allow early detection of any signs of hepatotoxicity.

For mild liver disease (e.g., non-alcoholic fatty liver disease), acetaminophen can be used at standard dosages, typically 500-1000mg every four to six hours as needed. However, the total daily dose should not exceed 4,000mg.

For severe liver disease (e.g., cirrhosis), dosing should be reduced (2-3g/day), and using the lowest effective dose is generally advisable for the shortest duration.

## Conclusions

When used cautiously and within recommended dosages, acetaminophen remains a valuable option for pain and fever in individuals with liver disease. Acetaminophen should be considered a safe first-line analgesic in patients with liver disease. Healthcare providers should carefully evaluate each patient's liver function, choose appropriate dosing regimens, and closely monitor liver function during treatment. Patients, in turn, should be educated about the risks and benefits of acetaminophen use and advised to seek medical attention promptly if they experience any signs of liver dysfunction.
